# Regeneration of the lung: Lung stem cells and the development of lung mimicking devices

**DOI:** 10.1186/s12931-016-0358-z

**Published:** 2016-04-23

**Authors:** Kim A. A. Schilders, Evelien Eenjes, Sander van Riet, André A. Poot, Dimitrios Stamatialis, Roman Truckenmüller, Pieter S. Hiemstra, Robbert J. Rottier

**Affiliations:** Department of Pediatric Surgery, Erasmus Medical Center-Sophia Children’s Hospital, PO Box 2040, 3000 CA Rotterdam, The Netherlands; Department of Pulmonology, Leiden University Medical Center, PO Box 9600, 2300 RC Leiden, The Netherlands; Department of Biomaterials Science and Technology, University of Twente, MIRA Institute for Biomedical Technology and Technical Medicine, Faculty of Science and Technology, P.O Box 217, 7500 AE Enschede, The Netherlands; Department of Complex Tissue Regeneration, Maastricht University, Faculty of Health, Medicine and Life Sciences, MERLN Institute for Technology-Inspired Regenerative Medicine, PO Box 616, 6200 MD Maastricht, The Netherlands

**Keywords:** Lung, Stem cells, Regeneration, Tissue engineering, Lung mimics

## Abstract

Inspired by the increasing burden of lung associated diseases in society and an growing demand to accommodate patients, great efforts by the scientific community produce an increasing stream of data that are focused on delineating the basic principles of lung development and growth, as well as understanding the biomechanical properties to build artificial lung devices. In addition, the continuing efforts to better define the disease origin, progression and pathology by basic scientists and clinicians contributes to insights in the basic principles of lung biology. However, the use of different model systems, experimental approaches and readout systems may generate somewhat conflicting or contradictory results. In an effort to summarize the latest developments in the lung epithelial stem cell biology, we provide an overview of the current status of the field. We first describe the different stem cells, or progenitor cells, residing in the homeostatic lung. Next, we focus on the plasticity of the different cell types upon several injury-induced activation or repair models, and highlight the regenerative capacity of lung cells. Lastly, we summarize the generation of lung mimics, such as air-liquid interface cultures, organoids and lung on a chip, that are required to test emerging hypotheses. Moreover, the increasing collaboration between distinct specializations will contribute to the eventual development of an artificial lung device capable of assisting reduced lung function and capacity in human patients.

## Background

Although the lung has a low rate of cellular turnover during homeostasis, it has a remarkable ability to regenerate cells after injury [1]. Disruption of this regeneration potential is the cause of several lung diseases. Therefore, understanding the underlying mechanisms of the regenerative capacity of the lung offers potential in identifying novel therapeutic targets. Much can be learned from studies on lung development as processes involved in the differentiation of cell lineages during development are recapitulated during repair [2]. Recent advances in the identification of new cell markers, the analysis of cell fate by in vivo lineage tracing experiments, the use of embryonic and induced pluripotent stem cells, and improvements in organoid cultures have increased the knowledge about the presence of potential stem cells in the lung [3–6]. The goal of this review is to survey the latest developments in endogenous lung regeneration and bioengineering of lung models for therapeutic applications in the future. We will first provide an overview of the latest insights in lung progenitor cells and their potential to differentiate into lung epithelial cells, which is of interest for the in vivo regeneration of lung tissue. Next, we will discuss the plasticity of the different epithelial cells in the lung and their potential to contribute to epithelial regeneration. Finally, we will highlight the possible clinical applications of this knowledge, focusing on different populations of stem cells, lung mimics and tissue engineering.

## Potential epithelial stem cells of the lung

**Fig. 1:**
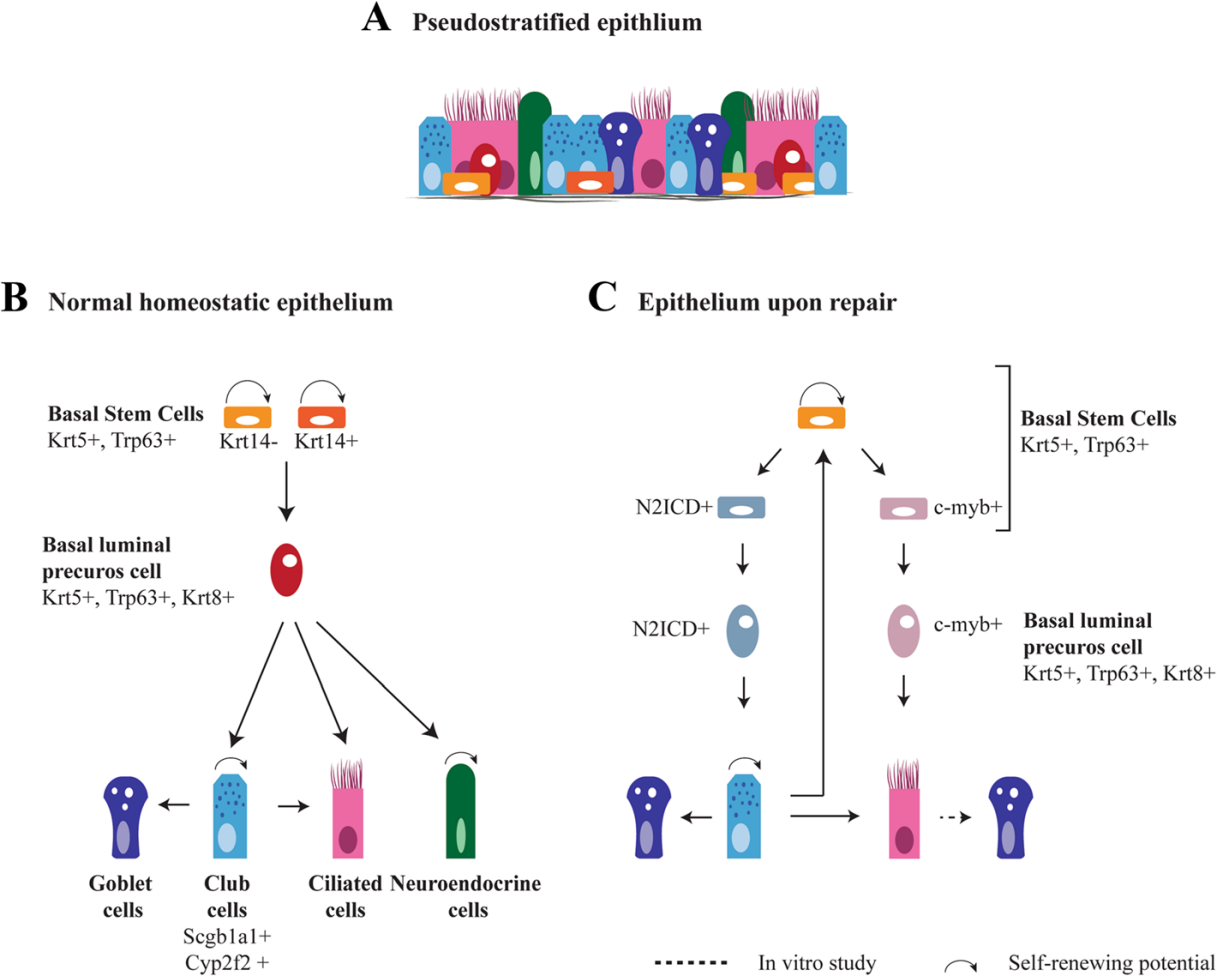
Regeneration of pseudostratified airway epithelium of the lung. **a** The airways are lined by a pseudostratified epithelium consisting of secretory cells (goblet and club cells), ciliated cells, neuroendocrine cells and basal cells. Goblet cells are abundant in the human epithelium, but are rare in mice. **b** The relationship between the different epithelial cells during normal homeostasis. The basal cells are progenitor cells of the pseudostratified epithelium which are heterogeneous for the expression of Krt14. The basal cell becomes a Krt8 positive luminal precursor cell before further differentiation. A basal cell differentiate into secretory cells and neuroendocrine cells under homeostatic conditions. Neuroendocrine cells are also capable to self-renew [162]. Scgb1a1^+^ secretory cells are a self-renewing population and can give rise to ciliated cells. In homeostatic epithelium, there is a very low turnover of cells. It is likely that the dividing secretory cell population is sufficient to regenerate ciliated cells in homeostatic condition. However, their generation from basal cells is not excluded. Upon allergen exposure, secretory cells are the main source of goblet cells [163], but it is unknown whether basal cells can directly differentiate into goblet cells. (C). Upon depletion of luminal cells by SO_2_exposure, basal cells proliferate and subdivide into two populations, N2ICD and c-myb positive, respectively, differentiating into secretory and ciliated cells. After the loss of basal cells, secretory cells (de)differentiate into functional progenitor basal stem cells. In a normal pseudostratified epithelium, only a few scattered goblet cells are present. Increases in goblet cells are observed upon immune stimuli and in diseases like COPD. Lineage tracing studies show that goblet cells can arise from Scgb1a1^+^ secretory cells and recently a trans-differentiation of foxj1^+^ ciliated cells to goblet cells was observed upon smoke exposure in culture

**Fig. 2:**
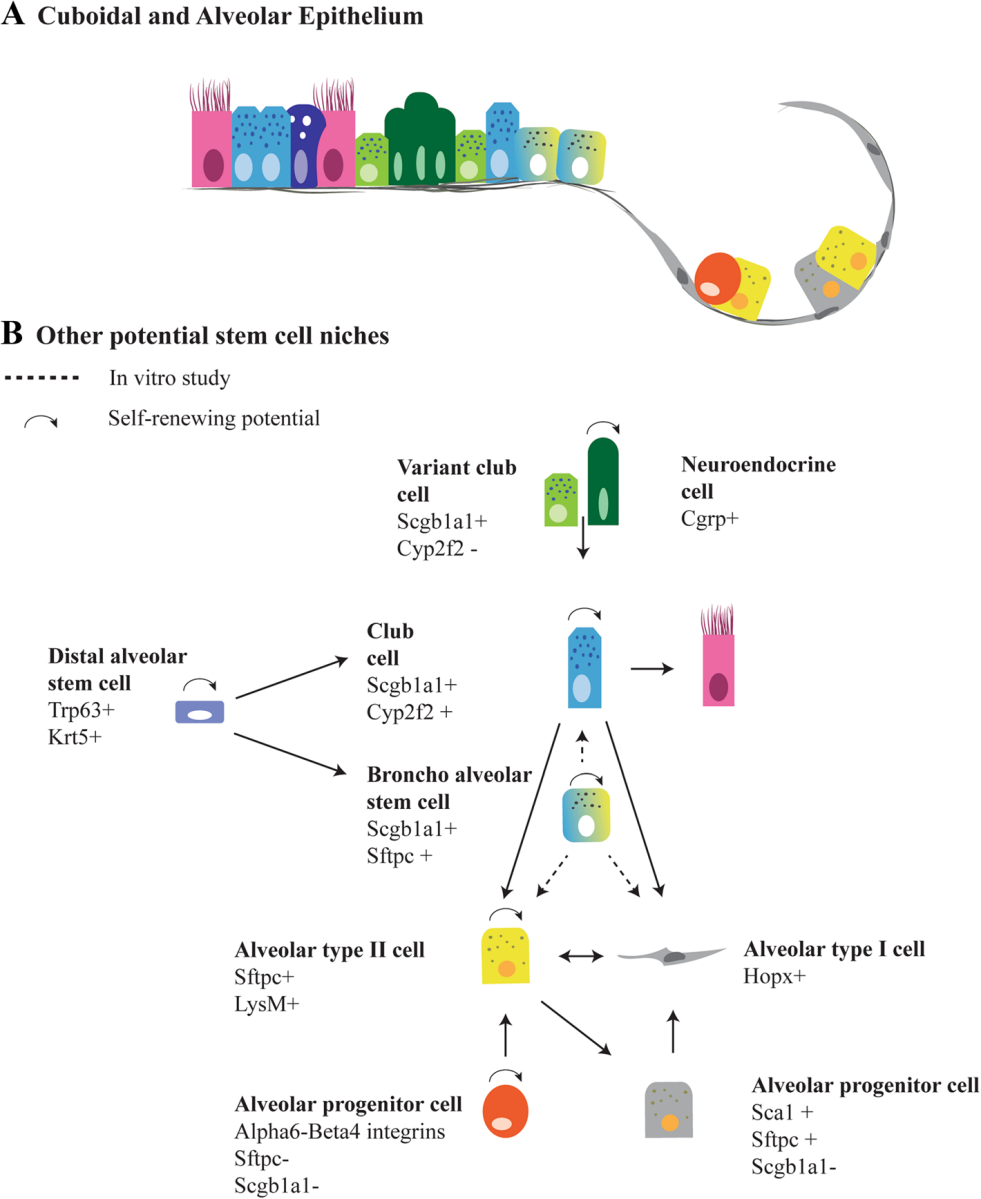
Regeneration of distal and alveolar airway epithelium after injury. **a** The small airways lack basal cells and consist of cuboidal epithelium, containing secretory and ciliated cells, as well as clusters of neuroendocrine cells. The cuboidal epithelium passes into a broncho-alveolar duct junction which is the niche of broncho-alveolar stem cells. The alveolar epithelium consists of alveolar type I, type II cells and alveolar progenitor cells. **b** Variant club cells (Cyp2f2^−^) are a variant of secretory cells that survive naphtalene injury and give rise to cyp2f2^+^ club cells. Lineage tracing of Cgrp^+^ cells showed that after depletion of club cells by naphtalene injury neuroendocrine cells contribute to the regeneration of these cells. At the broncho-alveolar duct junction, broncho-alveolar stem cells were isolated and shown to differentiate into bronchiolar and alveolar lineages in culture (dashed lines). Scgb1a1^+^ cells have the potential to form alveolar type I and type II cells after bleomycin injury, but not after hyperoxia-induced injury (dashed line). AT-II cells can self-renew and differentiate to AT-I cells. After pneumonectomy, a contribution of AT-I cells to regenerate AT-II cells was observed. An alveolar progenitor cell expressing α6-β4 integrins can regenerate AT-II cells after injury. Yet another cell type was identified expressing Sca1^+^ arising from AT-II cells and regenerating AT-I cells. Distal alveolar stem cells appear after severe injury and give rise to secretory and alveolar cells

Different subsets of epithelial cells and potential stem cell niches have been identified in the lung. The airways of the human lung are lined by a pseudostratified epithelium made up of basal cells, secretory cells (Scgb1a1^+^ club cells and goblet cells), ciliated cells and neuroendocrine cells (Fig. 1a). The trachea of the mouse, a frequently used model in research, has a similar architecture as the human airways. In human airways, basal cells decrease in frequency from the large to the distal airways [7]. The airways of the mouse and the respiratory smallest bronchioles of the human lung are covered by a cuboidal epithelium. This epithelium lacks basal cells and contains ciliated cells, secretory cells and neuroendocrine cells that are usually clustered in neuroendocrine bodies (NEBs) (Fig. 2a) [8]. The alveoli of both human and mouse are composed of two functional distinct cell types, flat and extended alveolar type I (AT-I) cells to allow gas exchange and cuboidal alveolar type II (AT-II) cells for surfactant protein production and secretion (Fig. 2a) [2, 9]. New emerging technologies, such as single cell RNA-sequencing and proteomic analysis, revealed molecular signatures that hint at different subpopulations of type I and type II cells. It remains to be seen whether such signatures reflect functionally different cell types, or that it represents similar cells at physiologically or metabolically different phases. However interesting, this is not the focus of this review, and therefore we only refer to the current literature [10–12].

### Basal-like stem cells: the stem cells of the epithelium

Basal cells are being characterized by the expression of Trp63, Ngfr, podoplanin (Pdpn, also known as T1α), GSIβ4 lectin and cytokeratin5 (Krt5). They have the capacity to self-renew and to form secretory and ciliated cells (Fig. 1b) [13–15]. Notch signaling plays a major role in determining the differentiation of basal cells to either the secretory lineage or the ciliated lineage [15–17]. A small subset of the basal cells (<20 %) expresses Krt14 under homeostatic conditions. These cells are thought to be a self-renewing population involved in maintenance of the Krt5^+^ basal cell population. This proportion is highly increased and becomes multipotent after naphthalene-induced depletion of secretory cells [18, 19]. Lineage tracing studies show that Krt14^+^ cells can directly regenerate secretory and ciliated cells [18, 20]. Recently, two distinct populations of basal cells were identified in the adult lung using long-term lineage tracing experiments and single-cell gene expression profiling: basal stem cells (BSCs) and basal luminal precursor cells (BLPCs). Both cell types are Krt5^+^ and Trp63^+^ with rare detection of Krt14, indicating that Krt14 is not a robust marker for stem cell identity [21]. However, the rapid up-regulation of Krt14 post-injury suggests that Krt14 may be an important marker to identify activated stem cells in the regenerating epithelium. Within homeostatic conditions, BSCs divide via asymmetric division to produce one new BSC and one BLPC, which can further differentiate into a neuro-endocrine and secretory cell (Fig. 1b). The BLPCs have a low or negligible rate of self-amplification, lack any overt signs of differentiation, and are distinct from BSCs by their expression of Krt8 [21]. This model is consistent with a previous observation in human basal cells addressing the potential of individual basal cells to self-renew and differentiate [22]. Additionally, the emergence of a Krt5^+^/Krt8^+^ parabasal cell population, which have comparable characteristics as the previously described BLPCs, was shown to be controlled by active Notch3 signaling [16]. Notch3^−/−^ mice showed an increase in basal cells and parabasal cells, but not in multiciliated and secretory cells, suggesting that Notch3 is involved in restricting the expansion of the basal and parabasal population [16]. Interestingly, binding of the transcription factor Grainyhead-like 2 (Grhl2) to the promotor region of Notch3 was observed, suggesting a role for Grhl2 in the transcription of Notch3 [23]. BSC-specific ablation of Grhl2 showed only a decrease in the number of ciliated cells, but no other changes in the morphology of the epithelium [24]. Whether Grhl2 is important in the Notch3 dependent regulation of the BSC and parabasal cell population still has to be explored. Krt8^+^/Krt5^+^ double positive cells were previously identified in mice as a marker for progenitor cells upon regeneration following injury induced by reactive oxygen species and sulfur dioxide (SO_2_) [15, 25]. Interestingly, using the SO_2_ injury model, it was observed that Trp63^+^ basal cell populations segregate in subpopulations prior to the formation of the Krt8^+^ progenitor cell. These dividing Trp63^+^ basal stem cell populations are either N2ICD^+^ (the active Notch2 intracellular domain) cells that differentiate into mature secretory cells, or c-myb^+^ cells that differentiate into ciliated cells (Fig. 1c) [26]. This specific segregation of progenitor cells was not found in homeostatic epithelium, which indicates that post-injury mechanisms may lead to different subsets of progenitor cells compared to the homeostatic epithelium [26]. A new study shows Trp73 as a regulator of ciliated cell differentiation, which expression was observed in terminally differentiated ciliated cells as well as in Trp63^+^ basal cells. This indicates a direct transition from basal cell to ciliated cell as well as a segregation of epithelial cell fate at the basal cell level [27]. The role for Trp73 in response to damage and the trigger that is responsible for a Trp73^+^ basal cell to initiate ciliated cell differentiation is not yet studied. This would be essential in understanding the role of Trp73 in the Trp63^+^ basal cell population.

Clusters of Trp63^+^/Krt5^+^ cells, called distal alveolar stem cells (DASCs), are present in the distal airways after H1N1 influenza virus infection and have the capacity to replace injured alveolar cells (Fig. 2b) [28, 29]. Despite sharing similarity in markers, the tracheal basal stem cells (TBSCs) and DASCs show different fates in culture and in vivo transplantation. The TBSCs give rise to more proximal epithelium both in culture and in vivo, while the DASCs can form alveolar spheres in vitro and give rise to alveolar cells and secretory cells in vivo [29]. Krt5 lineage tracing studies concluded that these cells were not present before infection and were generated as a response to injury [29]. In addition to this finding, Vaughan and colleagues proposed a lineage negative epithelial precursor (LNEP) cell expressing Trp63^+^ and Krt5^+^ that helps to regenerate the alveoli after bleomycin injury. Transcriptional profiling of these cells indicate a very heterogeneous population suggesting that different cell types are present in the Trp63^+^/Krt5^+^ population [30]. Moreover, active Notch signaling was required to activate Trp63^+^/Krt5^+^ expression in LNEPs and active Notch prevents the further differentiation into AT-II cells [31]. This suggests that the hyperactive Notch signaling observed in lung diseases possibly contributes to failure of regeneration. In conclusion, basal cells can function as tissue-specific stem cells of the airway epithelium, but the heterogeneity in the population of basal cells is not yet completely understood. Since the identification of different subsets of basal cells is studied using lineage-tracing studies in mice, validation of these subsets of basal cells in human lung is of importance. Differences in progenitor populations are found in homeostatic epithelium compared to damaged epithelium. This suggests that in response to injury, molecular mechanisms are triggered that lead to the appearance of different subsets of epithelial progenitor cells, perhaps derived from one general homeostatic basal cell. Currently, signaling pathways are being identified that influence the expansion of basal cells and differentiation into specific cell types, but the precise underlying molecular mechanisms still need to be identified (Table 1). Furthermore, it is increasingly recognized that basal cells not only contribute to tissue repair, but are also a target for respiratory pathogens and contribute to host defense against infection [32]. Further studies, including those aimed at identifying subsets of basal cells that display these properties, are needed to better understand the link between this immune basal cell response and repair of the epithelium.

**Table 1:** Overview basal-like stem cell populations

Cell type	Subtypes	Differentiation potential	Signaling Cues
(Tracheal) Basal Stem Cells	Trp63, Krt5, Krt14^+/−^	Self-Renewal	Notch [25], Hippo signaling [44]
	Trp63, Krt5, Krt8	Basal Luminal Precursor Cell	Notch 3 signaling [16], Grhl2 [24]
		Neuroendocrine	Notch1 [164, 165] and Hes1 [166]
	Trp63, Krt5, N2ICD	Club	Notch^high^ signaling [15], Notch1 [167], Notch2 [164]
	Trp63, Krt5, c-Myb/Trp73	Ciliated	Notch^low^ signaling [15], Notch 1 and 2 [164]
Distal Alveolar Stem Cells ^a^	Trp63, Krt5	Self-Renewal	Increased Notch activity, Notch1 [31]
		AT-II	Inhibition of Notch [31]
		Club	

^a^Only observed after H1N1 influenza virus infection or bleomycin induced injury, *AT-II* Alveolar Type II Cells

### Other epithelial progenitor cells

Basal cells are not the only identified multipotent cells in the lung (Table 2). Variant club cells, a subset of secretory cells that are positive for secretoglobin family 1a member 1 (Scgb1a1) and negative for Cyp2f2, have been shown to self-renew and to differentiate into Cyp2f2^+^ secretory cells after naphthalene injury [3, 33, 34]. Interestingly, another subset of Scgb1a1^+^ cells co-expressing the AT-II marker surfactant protein C (Sftpc) was shown to differentiate into bronchiolar and alveolar lineages in vitro. These cells were called broncho-alveolar stem cells (BASCs) and are located at the broncho-alveolar duct junction (BADJ) (Fig. 2b) [35]. However, conflicting results are reported based on lineage tracing of Scgb1a1^+^ cells after lung injury. Scgb1a1^+^ cells differentiate into alveolar epithelial cells after influenza and bleomycin-induced injury, but not after hyperoxia-induced alveolar injury [34, 36]. This contradiction could result from different subsets of cells being labeled by the Scgb1a1-driven Cre driver, or from the activation of different pathways by hyperoxia and bleomycin. Cell-specific lineage tracing tools are required to give more clarity about the potential of BASCs and the variant club cells.

**Table 2:** Other potential epithelial stem cells

Cell type	Marker genes	Differentiation potential	Hallmarks
Variant Club Cells	Scgb1a1^+^, Cyp2f2^−^	Club, Ciliated	Located near NEBs
		Ciliated	Survive Naphthalene injury
Broncho-Alveolar Stem Cells	Scgb1a1^+^, Sftpc^+^	AT-II, Ciliated	Wnt signaling induces proliferation BASC [168]
			Located at BADJ
Itgα6^+^, Itgβ4^+^Alveolar progenitor	Scgb1a1^−^, Sftpc^−^, Itgα6^+^, Itgβ4^+^	AT-II, Club	Located at BADJ and Alveolar wall
AT-II	Sftpc^+^, LysM	Sca1^+^, Sftpc^+^ AT-I progenitor cell	EGF induced proliferation [37]
		AT-I	Wnt dependent conversion to AT-I [41]

*NEBs* Neuroendocrine Bodies, *BADJ* Broncho-Alveolar Duct Junction, *AT-I/II* Alveolar-Type I/II cells

Different alveolar progenitors and associating markers have been identified in response to lung injury and are summarized in Fig. 2b. AT-II cells expressing Sftpc are capable of self-renewal and a small fraction of mature type II cells can differentiate into AT-I cells in homeostasis and after injury [37, 38]. Besides the progenitor potential of AT-II cells, another progenitor subpopulation for alveolar epithelial cells has been identified. These cells co-express α6 and β4 integrins, but lack expression of Scgb1a1 or Sftpc. They respond to lung injury and can differentiate into AT-II cells and club cells. These cells reside in the alveoli as well as in the BADJ and their differentiation potential in vivo is most likely restricted by their niches [39]. Furthermore, a distinct population of Sca1^+^/Sftpc^+^ AT-II cells appeared at the onset of repair after infection of the lung by *Pseudomonas aeruginosa* intratracheal instillation [40, 41]. Most of these cells were negative for β4 integrin, Trp63 and Scgb1a1, separating them from respectively other distal progenitor cells and BASCs [28, 35, 39, 41]. Lineage tracing experiments showed that Sca1^+^ AT-II cells may arise from Sftpc^+^/Scgb1a1^−^ cell and further differentiate into AT-I cell (Fig. 2b). This conversion of Sca1^+^ AT-II cells to AT-I cells depends on an active Wnt/β-catenin pathway [42]. Taken together, several populations are being marked as progenitor cells and the activity of subsets of progenitor populations seems to depend on their niches and kind of epithelial damage. The current challenge is to elucidate whether the different progenitor cells are indeed different cells, or if these cells are variants of a single precursor cell that are induced by different damaging agents. Single-cell RNA sequencing of the developing distal lung epithelium has helped in defining more precisely the different types of (progenitor) cells in the distal region of the developing lung [12]. A similar approach during regeneration of the proximal and distal lung epithelium might provide additional clues on the heterogeneity of epithelial cells upon repair.

## Plasticity of the lung

Further complexity and challenges in lung regeneration are generated by the plasticity of differentiated cells (Table 3). Independent studies have pointed at the potential of Scgb1a1^+^ secretory cells to dedifferentiate into Trp63^+^/Krt5^+^ basal cells upon depletion of the basal cell lineage or after damage of the lung epithelium [14, 43]. These dedifferentiated basal cells have the full capacity to redifferentiate into ciliated or secretory cells (Fig. 1c). The Hippo pathway and its down-stream effector Yap are required for the dedifferentiation of secretory cells [44]. Moreover, Yap has been shown to regulate stem cell proliferation and differentiation during normal epithelial homeostasis and regeneration upon injury in the adult lung [44, 45]. Further research showed that the nuclear-cytoplasmic distribution of Yap is important in the differentiation of adult lung epithelium and during development [16, 46]. Thus, Hippo signaling may be important in stimulating regeneration of the pseudostratified epithelium by controlling basal stem cell differentiation as well as luminal cell plasticity.

**Table 3:** Plasticity of differentiated cells

Cell type	Marker genes	Differentiation potential	Signaling Cues
Club cells	Scgb1a1^+^, Cyp2f2^+^	Basal	Hippo pathway [44]
		Ciliated	*Unknown*
		Goblet	IL-13 exposure [169]
AT-I	Hopx	AT-II	Modulated by TGF-β signaling [49]
			Proximal cell fate by overexpressing Sox2 [51]
AT-II cells	Sftpc^+^, LysM	Proximal cells	Ectopic Sox2 expression [50]
Ciliated cells	TubIVa, Foxj1	Goblet	IL-13 exposure [47]

*AT-I/AT-II* Alveolar type-I/II Cell

Differentiation of Foxj1^+^ ciliated cells to mucus-producing goblet cells was observed in human primary bronchial epithelial cell culture after exposure to IL-13, an important mediator in asthma [47]. Interestingly, this plasticity was not confirmed by a Foxj1^+^ lineage tracing study in mice using an ovalbumin-induced injury model [48]. Either the difference of damage to the epithelium, smoke versus ovalbumin, or the use of different species could account for the different outcomes.

Previous lineage tracing studies using lysozyme M as marker for mature AT-II cells already demonstrated that AT-II cells can differentiate into AT-I cells [37]. More recently, a plasticity AT-I cells after pneumonectomy has been shown. To regenerate the alveoli, Hopx^+^ AT-I cells proliferate and differentiate into Sftpc^+^ AT-II cells (Fig. 2b) [49]. The formation of AT-II cells from Hopx^+^ AT-I cells in organoid culture seems to be modulated by TGF-β signaling [49]. These results suggest a bi-directional transition between the two types of mature alveolar cells. However, after pneumonectomy the contribution of AT-I cells to regenerate AT-II cells is small (~10 %). Vice versa, approximately 16 % of regenerated AT-I cells are derived from Sftpc^+^ AT-II cells, indicating that other cell sources also contribute to re-alveolarization [49]. Thus, strategies for regeneration of lung epithelium in disease, includes targeting of progenitor cell populations and activating the plasticity or fate of differentiated lung cells. Signaling cues to induce endogenous lung regeneration are starting to be identified and might be targets for disease therapies in the future. In line with initiating differentiation through signaling, it has been demonstrated that conversion of a specific cell type can be induced by changing the expression of a single protein. Ectopic expression of Sox2 in AT-II cells changed its alveolar cell type to a more proximal cell fate expressing Scgb1a1 and Trp63, even though the cells remained in the niche for distal cells [50]. A similar approach was used to show the plasticity of AT-I cells, where overexpression of Sox2 was sufficient to reprogram AT-I cells towards a proximal airway cell fate with expression of Trp63 [51]. The differentiation potential and plasticity of the lung epithelial cells as described in the above sections are illustrated in Figs. 1 and 2 to show the complexity of the cells involved in regenerating the lung epithelium.

## Regenerative medicine

### Drugs to induce lung regeneration

Different signaling pathways are involved in either maintaining a quiescent homeostatic or inducing a proliferating regenerating epithelium [3]. Signaling consists of cross-talk and feedback loops between epithelial cells but also between epithelial and mesenchymal cells. Such interplay between mesenchymal and epithelial cells is for example important in Hedgehog (Hh) signaling. In the adult lung, Hh signaling balances between stimulating proliferation and quiescence. In the homeostatic lung Hh signaling is active to maintain quiescence, however upon injury Hh signaling is inhibited to stimulate epithelial proliferation [52]. A shift in the balance can lead to failure of repair but can also play a role in promoting tumorigenesis [52, 53]. Several pathways involved in lung development and regeneration are relevant in lung disease, and drugs that either inhibit or induce these pathways could have a beneficial effect for patients. Recently, it was shown that deletion of Notch3 leads to an expansion of basal cells, a hallmark of smokers and individuals with chronic obstructive pulmonary disease (COPD) [16, 54, 55]. Interestingly, Notch3 down-regulation was observed in smokers and in COPD lung, making it a potential target for controlling the balance between basal and luminal cells [16, 56] Candidate pathways for targeting in COPD include Hedgehog signaling, Notch signaling, the retinoic acid pathway and the transforming growth factor-β (TGF-β) pathway [57]. The TGF-β pathway, as well as bone morphogenetic proteins (BMPs), growth differentiation factors and activins are also linked to asthma and these pathways could be potential drug targeting candidates [58]. In COPD there is mucus hypersecretion, and there are several ongoing studies that examine the effect of already marketed drugs on the production and secretion of mucus in COPD models [59]. Recently, it was shown that interference of Notch signaling with specific antibodies against the ligands Jag1 and Jag2 results in an increase in ciliated cells at the expense of club cells [60]. Moreover, jagged inhibition also reversed goblet cell hyperplasia, which could potentially be important in COPD patients to reduce the mucus production and to increase clearance by the ciliated cells. Fibroblast growth factors (FGFs) also play a role in regeneration of several tissues including the lung [61]. FGF1 and FGF2 are thought to play a role in the protection of epithelial stem cells and lung maintenance, and are linked to pulmonary hypertension. FGF1 is also thought to play a role in idiopathic pulmonary fibrosis. FGF7 and FGF10 are involved in lung regeneration and several different injury models show that these FGFs are important for repair of the lung. Several recombinant FGFs (FGF1, FGF2) and truncated forms of FGFs (FGF7, FGF10) are already used in clinical applications, like angiogenic therapies, coronary heart disease and treatment of ulcers [61]. Although these therapies are not yet available for lung diseases, there may be some future perspectives, either in inducing or inhibiting pathways involved in disease or by activation of endogenous lung progenitor cells.

## Stem cells

Stem cells are functionally characterized by their undifferentiated state and their properties of self-renewal and pluripotency to become specialized cells. Because of these characteristics, they are appealing to be used for the regeneration of damaged tissue. A distinction can be made between embryonic stem cells (ESCs) and adult stem cells. ESCs are derived from the inner cell mass of the blastocyst and these cells have the ability to differentiate into ectodermal, mesodermal and endodermal cell types. Human ESCs could be useful to study early embryonic development, for cell replacement therapy, to study disease pathways, and for drug discovery, although ethical and therapeutic issues hamper the use of these cells. Besides ESCs, several types of adult stem cells throughout the human body exist, like hematopoietic stem cells, intestinal stem cells, mammary stem cells, olfactory stem cells, mesenchymal stem cells, endothelial stem cells, and neural stem cells. Adult stem cells are also capable of self-renewal and may differentiate into several cell types, but their differentiation potential is more restricted [62–64]. As indicated, there is accumulating evidence that differentiated cells show more plasticity than previously thought. Moreover, the number of different progenitor cells in the lung is higher than previously expected, depending on the type of injury or disease.

### Induced pluripotent stem cells

In 2006, the group of Yamanaka introduced a method to generate cells with properties similar to ESCs [65]. These so-called induced pluripotent stem cells (iPSCs) are somatic cells that are reprogrammed into a multipotent stem cell-like stage using only four different factors: Oct4, Sox2, cMyc and Klf4 (Yamanaka factors) [64]. Culturing these cells under distinct conditions induces several specialized cell types. iPSCs can be used for numerous applications, like disease modeling, regenerative medicine, drug discovery, and toxicity studies [64, 66].

The lung is a very complex organ that consists of many different specialized cell types, which makes it challenging to generate human airway and alveolar epithelial cells from iPSCs. First, definitive endoderm should be derived from human iPSCs (hiPSCs), followed by generation of anterior foregut endoderm [67]. From this anterior endoderm, lung endoderm can be derived, which can subsequently be guided towards bronchial progenitor cells (Sox2^+^) or alveolar progenitor cells (Sox9^+^), and finally towards bronchial or alveolar epithelial faith [64]. Several studies have shown the differentiation of ESCs and iPSCs into AT-II cells [68–73]. Other groups have shown the differentiation of iPSCs into multiciliated cells [74], mature airway epithelium expressing functional CFTR protein [75], multipotent lung and airway progenitors [76], purified lung and thyroid progenitors [77], purified distal lung alveolar epithelium [78], lung and airway epithelial cells [79], and lung and airway progenitor cells [80]. An overview of these differentiation protocols is given by Ghaedi and co-workers, although optimization is clearly required before these cells may be used in clinical applications [64].

iPSCs may be used for the generation of patient-specific disease models and (large scale) drug screening, as shown for example with cells derived from patients suffering from cystic fibrosis [81]. A more clinical use of iPSCs in lung disease therapy is not yet approved and more knowledge is necessary before this will be applicable [82].

### Mesenchymal stem cells

Mesenchymal stem/stromal cells (MSCs) are adult stem cells that have the potential to differentiate into cells derived from the mesoderm lineage. MSCs were first derived from bone marrow, but many other sources are reported, including umbilical cord blood, placenta, skin, liver and brain [83, 84]. MSCs refer to a heterogeneous population of cells, making it difficult to isolate them. Therefore, MSCs are defined by a number of criteria based on the expression of specific cell surface antigens and their functionality. Cells should express CD75, CD90 and CD105, but not CD34, CD45, HLA-DR, CD11b, CD19 and CD14. MSCs should be capable to differentiate into chondrocytes, osteoblasts and adipocytes, and should adhere to plastic for stable cell culture [62, 83–85]. Recent studies have shown that MSCs may differentiate in other cell types, including lung cells, although this is still controversial [86, 87]. It has been reported that MSCs can also be isolated from the lung. Martin et al. reported the isolation of MSCs from tracheal aspirates of neonates and from adult broncho-alveolar lavage [88]. More recently, Gong and co-workers isolated lung resident MSCs and showed that these cells have the potential to differentiate into AT-II cells [89]. MSCs derived from other sources than the lung can also be differentiated into alveolar epithelium. These alveolar cells were generated from MSCs derived from human umbilical cord blood by culturing them in lung-specific differentiation media [87].

There are many completed and ongoing clinical trials using MSCs for applications in the nervous system, heart, liver and kidney. In lung disease, therapies with MSCs could be useful in bronchopulmonary dysplasia (BPD), COPD, acute respiratory distress syndrome and idiopathic pulmonary fibrosis [62, 83, 85, 90, 91]. However, given the low percentage of engraftment of the instilled MSCs as demonstrated in animal models, it is very likely that the beneficial effects of MSC therapy are not due to the differentiation potential of MSCs itself, but rather due to paracrine and immunomodulatory effects [83, 92–94].

### Endothelial progenitor cells

There are two different subsets of endothelial progenitor cells (EPCs), proangiogenic hematopoietic cells and endothelial colony-forming cells (ECFCs) [95]. Proangiogenic hematopoietic cells are derived from the bone marrow and are involved in vascular repair. It is thought that these cells circulate to injury sites and there facilitate formation of new vessels using paracrine mechanisms, but lack direct vessel-forming ability. ECFCs are rare circulating blood cells that have the potential to generate cells that express genes from the endothelial lineage. They also have the potential to form blood vessels in vivo [95]. There is increasing evidence that EPCs are involved in several lung diseases, including COPD, BPD and pulmonary hypertension. Several lung injury animal models have shown (partial) reversal of the induced phenotype by systemic administration of EPCs, including improvement of pulmonary function and repair of the alveolar and vascular structure of the lung [96–98]. These therapeutic effects could be caused by structural conditions of the cells, by paracrine effects or by a combination of both [82]. The interaction between the pulmonary vasculature and the airways is important for proper growth and regeneration of the lung (reviewed in [99]). This was recently supported by the identification of endothelial derived angiocrine signals promoting alveolar regeneration after pneumonectomy [100, 101]. The interactions between the vasculature and epithelial cells upon repair are still elusive, but the identification of signaling molecules, like stromal cell-derived factor-1 (SDF-1), may be important for potential therapies. Systemic administration of EPCs has shown to be beneficial in patients with primary pulmonary hypertension [102, 103]. Several pre-clinical and clinical trials are ongoing to test the potential of using EPCs in lung disease therapies [82].

Besides the stem cells mentioned in this section, there are also endogenous lung progenitor cells that were discussed in previous sections. All these different stem/progenitor cells are potentially targets for therapeutic strategies. While MSCs and EPCs could be effective because of their paracrine effects, iPSCs could be useful in the development of lung mimics and tissue engineering. Pathways involved in differentiation of lung progenitor cells to other cell types and plasticity of these cells, could be induced or inhibited by medication to induce lung regeneration.

## Lung mimics

Most studies on cell biology and tissue regulation are based on 2D cell-culture models. Although these models are valuable to answer specific scientific questions, it is clear that these models have limitations and fail to reconstitute the in vivo cellular microenvironment. Therefore, 3D cell-culture models were developed, which mimic a more realistic tissue- and organ-specific micro-architecture, although some aspects, including tissue-tissue interfaces and a mechanically active microenvironment are still missing. However, these models are very useful in patient-specific disease models, drug-screening and as a source of cells for transplantation [104].

### Air-liquid interface cultures

Air-liquid interface (ALI) cultures mimic a more realistic lung environment and make it possible for airway epithelial cells to proliferate and differentiate in vitro. Whitcutt et al. were the first to demonstrate mucociliary differentiation using ALI cultures [105]. Culturing human airway epithelial cells from patients, makes it possible to conduct patient-specific research and drug-screening, for example in cystic fibrosis and asthma [106, 107]. ALI cultures were also used to model the effects of smoke exposure on epithelial cells, which could be used to gain more insight in mechanisms involved in the pathogenesis of COPD [108, 109]. In 2015, a new computer-controlled ALI culture system was introduced in order to generate more stable and comparable cultures, which may be useful for large-scale toxicology studies [110].

### Organoids

The concept of stem cell-derived organoids has already been discovered in the 1950’s [111]. Organoid models use the pluri- or multi-potent properties of stem cells to differentiate into specialized cell types and to self-organize into a 3D structure with organ- or tissue-specific morphogenetic and histological properties [112–114]. Overviews of tissues and diseases modeled with organoids have been topics of recent reviews [113–115]. These tissues include intestinal buds, liver bud derivatives and retinal derivatives. In the intestine, single Lgr5^+^ stem cells can be isolated and grow into intestinal organoids [116]. Generation of lung organoids from one single stem cell have not been reported yet, but several studies have reported the generation of lung organoids derived from human pluripotent stem cells (hPSCs), primary respiratory cells and cell lines (reviewed in [117]). These organoids include trachea/bronchospheres [15, 118–120], bronchiolar organoids [121], bronchioalveolar organoids [120, 121], alveolospheres [38, 120, 121], branching structures [122–124], alveolar spheroids [125] and multi-lineage organoids [126]. In 2015, Dye et al. established a protocol to successfully generate lung organoids derived from hPSCs (embryonic and induced). hPSCs were first differentiated into anterior foregut spheroids, using ActivinA, BMP and TGF-β inhibitors. This anterior foregut endoderm was subsequently induced into a lung lineage by modulating FGF and Hedgehog signaling. In this way, the foregut spheroids gave rise to lung organoids. These organoids possess both proximal airway-like structures and immature alveolar airway-like structures and are globally similar to fetal human lung. These human lung organoids can be used to study lung development and regeneration [127]. Previously, tracheospheres were used to show the capacity of basal cells to self-renew and the potential to form secretory and ciliated cells [13]. Jain et al. used organoid cultures to show the potential of Hopx^+^ AT-I cells to form AT-II cells [49]. Furthermore, application of the Clustered Regularly Interspaced Short Palindromic Repeats/Crispr associated protein (CRISPR/Cas) system in organoid culture might be a method to identify important players in epithelial cell differentiation. Recently, this approach was used to identify the role of transcription factor Grhl2 in the differentiation of ciliated cells [24]. In the future, the loss or gain of function by manipulation of genes in culture, will lead to more insight in potential stem cell populations in the lung. Organoids are very useful to answer specific questions about lung development and regeneration, but so far they are not exposed to air, resulting in incomplete differentiation of adult airway cells. Furthermore, it does not allow to expose organoids to air pollutants such as toxic gasses and micro- or nanoparticles, making it impossible to use them to study the effects of air pollutants on the airway epithelium.

### Lung-on-a-Chip

**Fig. 3:**
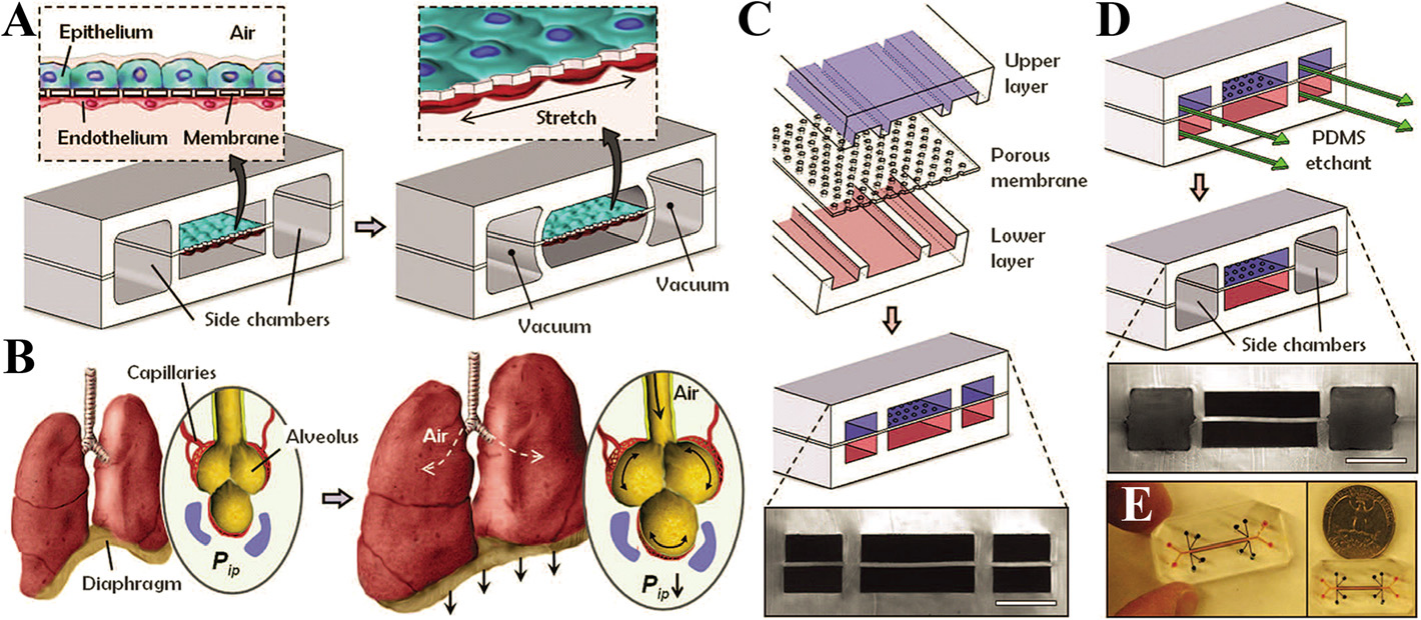
Example of a human breathing lung-on-a-chip microdevice. Lung-on-a-chip microfluidic device with compartmentalized microchannels to mimic a breathing lung (From Huh et al., “Reconstituting organ-level lung functions on a chip”, Science 2010; 328:1662–8. Reprinted with permission from the AAAS [100]). See original reference for detailed description of the figure. In brief, (**a**) indicates the creation of mechanical breathing movements causing mechanical stretch of the membrane, (**b**) shows the physiology of the normal breathing human lung, (**c**) and (**d**) show the assembly and etching of the microdevice, and (**e**) visualizes the actual size of the device

Organs-on-chips refer to bioengineered devices that mimic tissue properties and functions in a well-controlled environment [112]. Additionally, there are also (acellular) lung-mimicking microfluidic devices not specifically to study lung biology, but as respiratory assist devices or oxygenators [128]. Over the past decade, several micro-engineered organ models have been developed to study liver, kidney, intestine, and heart, among others [129]. The first lung-on-a-chip was introduced by Huh and co-workers, which mimicked the vascular-alveolar structure by using lung epithelial cells exposed to air on one side and pulmonary vascular endothelial cells exposed to flowing culture medium on the other side of a permeable synthetic membrane (Fig. 3, [130]). This model incorporated a microfluidics system and applied mechanical stress, and as such was capable of mimicking gas exchange. However, it also has some limitations, since it uses a flat 2D membrane, cell lines instead of primary cells, and lacks interstitial fibroblasts and alveolar macrophages [130–132]. In 2015, Stucki and colleagues reported a lung-on-a-chip with an integrated, bio-inspired respiration mechanism. This model used primary human pulmonary alveolar epithelial cells, which were co-cultured with endothelial cells and exposed to a 3D cyclic mechanical strain to mimic respiration [133]. The group of Blume developed a 3D model, consisting of an air-liquid interface culture of human primary airway epithelial cells in a microfluidic culture system. This system had a continuous exchange of fluids and mediators, thereby simulating the interstitial flow in the lung [134]. The power of using the lung-on-a-chip approach includes the possibility of connecting multiple devices, thereby creating a more realistic lung mimic by integrating microfluidics, stretch, curvature and primary cells. In addition to air-liquid-interfaces and mimicking stretch during in- and exhalation, the microfluidic approach allows to apply pressure and shear flow profiles both in alveoli and attached blood capillaries. Compartmentalized microfluidic systems make bioartificial/-engineered lung tissues also amenable for higher-throughput screening of the influence/impact of concentrations and mixtures of soluble factors in the blood/medium compartment, and of gases and particles in the air compartment

## Tissue engineering

Although the above described systems are rapidly evolving, a huge hurdle is the generation of whole tissues and organs. There are three important demands to successfully create tissues and organs: the source of cells, the type of scaffold, and the composition of the extracellular matrix (ECM). An appropriate mixture of cells should be used for the recapitulation of cell-cell interactions [135]. Appropriate scaffolds are necessary to obtain a 3D structure and can be either synthetic or biological, and biodegradable or non-biodegradable. In addition to the template three-dimensional structure, there is mechanical support and tissue instruction by engineered mechanical (e.g., through material or geometry-related matrix elasticity or stiffness), geometrical/topographical (e.g. through surface roughness or designed micro- or nanotextures) or (bio)chemical cues (e.g. RGD-adhesion moieties). An advantage of biodegradable scaffolds is that these are absorbed by the body. However, in the case of synthetic biodegradable scaffolds this may result in acidic degradation products causing inflammation in the surrounding tissue, e.g. when aliphatic polyesters like poly(lactic acid) are used [136]. Compared to synthetic scaffolds, biological scaffolds are more similar to the tissue or organ that they should substitute, although biological scaffolds may lack sufficient mechanical properties [137]. Different types of biological scaffolds can be used like collagen, Matrigel® and decellularized organs [137]. Decellularization of organs has to be done in a proper way to as much as possible preserve all components of the extracellular space/extracellular matrix components and their instructive properties. Several chemical, physical and enzymatical methods have been described to achieve this [62]. After decellularization, a process that does affect the extracellular matrix, the scaffold can be recellularized. Cells from different sources, as previously described, can be used for this purpose: embryonic, fetal or adult stem cells, autologous cells from the patient or iPSCs [62]. It is also possible to use allogeneic cells, e.g. in the case of transplantation of islets of Langerhans. There is also need for cells that are involved in vascularization and innervation, and cells with supportive, structural and barrier functions. Using autologous cells would be ideal to prevent rejection of the tissue-engineered organ in the patient, but could cause difficulties in the case of genetic or metabolic disorders [135]. Successful generation of tissue-engineered autologous bladders [138] and bio-engineered skin substitutes [139] have been reported as well as successful 3D bioprinting of several tissues and organs including multilayered skin, vascular grafts, heart tissue and tracheal splints [135, 140].

The structure and composition of the ECM should resemble that of embryonic organogenesis. It has been demonstrated that ECM signals are important to form pulmonary tissue structures in vitro [141]. Other signals, like cell-cell interactions, are also of importance to mimic the micro-environment of the organ [112, 142].

### Tracheal bioengineering

In patients with a tracheal defect of 50 % of total length in adults or 30 % in children, artificial tracheal grafting is required [143]. Several approaches for tracheal epithelial differentiation have been tested, including co-culturing of tracheal epithelial cells with fibroblasts or adipose-derived stem cells [144–146] and cell sheet engineering with tracheal epithelial cells [147, 148]. In spite of the controversies and success rate, Macchiarini et al. were the first group that transplanted a tissue-engineered airway [149]. The group of Steinke produced a bioartificial airway tissue using autologous primary cells to re-endothelialize and reseed a biological vascularized scaffold. After transplantation they observed complete airway healing and no evidence of tissue dedifferentiation [150]. Park and co-workers showed that human turbinated mesenchymal stromal cells cultured as intact sheets were able to differentiate into tracheal epithelium. These sheets were transplanted onto artificial grafts and tested in a rabbit model. After 1 month, regeneration of functional tracheal epithelium was observed [143]. Still, considerable problems are observed using tracheal grafts including failure to integrate and the formation of cartilaginous tissue [4, 62].

### Vascular bioengineering

Interactions between epithelium, mesenchyme and endothelium are necessary for proper lung development and regeneration. Blood vessels secrete angiocrine factors that are involved in these processes including KLEIP, HIF-2α, VEGF, BMP-4, FGF, MMP14, EET and TSP-1. Angiogenesis, the process where vessels are formed from a pre-existing network, is important for adult vascular homeostasis, regeneration and adaption. Angiocrine signaling is necessary for this process [99]. The important role of the vasculature is also recognized in tissue engineering. Ren et al. attempted to generate transplantable rat lung grafts by seeding epithelial and endothelial cells into the airway and vascular compartments of a decellularized lung scaffold from the rat. The major problem was poor vascular performance, causing incomplete endothelial coverage of the scaffold vessels. They optimized their protocol by co-seeding endothelial and perivascular cells which resulted in an endothelial coverage of 75 % [151]. Even during decellularization of lung scaffolds, vascularization is important to preserve the integrity of the scaffold [152]. Orlova and co-workers showed that it is possible to generate endothelial cells and pericytes from human PSCs. This could provide a source of patient-specific vascular cells used in vascular bioengineering [153].

### Whole lung bioengineering

Bioengineering of the whole lung is more complex than tracheal bioengineering due to the complexity of the lung. Lungs that are not suitable for transplantation can be decellularized and the scaffold can subsequently be used for seeding cells to regenerate the lung. It is still unknown which cell source is most suitable to repopulate the decellularized lung: MSCs, lung resident cells or a combination of both. Recently, it was shown that lung epithelial stem cells require co-culture with stromal cells to proliferate and differentiate. Fibroblasts have shown the highest efficiency in this support, and also the tissue origin of these cells gives varying patterns of support. Also, the use of FGFs and LIF-, ALK5- and ROCK-inhibitors activates proliferation and differentiation of quiescent lung stem cells [120]. Several methods were developed to decellularize lungs of rats, pigs, non-human primates and humans and to subsequently recellularize these scaffolds [154–160]. An overview of the currently available respiratory tract models, including the used cell sources and scaffolds, is reviewed by Nichols et al. [161].

## Conclusions

Knowledge about potential stem cells in the lung has markedly increased through various recent developments. One of the challenges will be to merge all the data from different species and obtained with various techniques into a simplified model of lung stem cells and their role in the normal and diseased lung. Furthermore, a comprehensive view of all the (un)differentiated cells is still missing, because our repertoire of cell specific markers is still inadequate to identify the various cell types. One concern is that the use of different markers in individual studies might lead to the misconception that several subpopulations of progenitor cells exist, whereas there may possibly be only a few. In the future, the increase of cell-specific markers combined with single-cell lineage tracing should improve the definition of different (stem) cell populations in the lung. Additionally, a universal and unambiguous biological read out system to test the quality and purity of lung stem cells is also unavailable. So far, different systems, such as ALI cultures, organoids and explants, are successfully employed to fill this gap, but this makes it cumbersome to compare the various studies. Together with ESCs, iPSCs, MSCs and EPCs, local lung stem or progenitor cells could be used for diverse clinical applications in the field of regenerative medicine. Current approaches to direct differentiation of stem cells, like iPSCs and MSCs, do generate lung-specific cells, but the specific lineages and the percentages of differentiated cells vary substantially. Therefore, optimization, improvement and expansion of the existing protocols is mandatory before clinical applications are possible. The manipulation of stem cells, like iPSCs, is required and useful for the development of lung mimics, for tissue engineering and for the generation of complete lung tissue. For tissue engineering applications, current scaffolds need to be improved or alternative suitable scaffolds need to be developed, which can be of synthetic and/or biological origin, and should contain appropriate ECM signals. Alternatively to bio-engineered lungs, specific pathways involved in differentiation of lung progenitor cells and plasticity of these cells may be targeted by novel compounds to induce their contribution to lung regeneration. Collectively, significant progress will be made through the interaction between very distinct scientific disciplines, such as developmental biology, biomedical engineering, and physics. These new and rapid developments in lung repair and regeneration offers a promising perspective for future patients with irreversible lung injury.

## Abbreviations

ALI: Air Liquid Interfase
AT-I: alveolar type I pneumocyte
AT-II: alveolar type II pneumocyte
BADJ: broncho-alveolar duct junction
BASC: broncho-alveolar stem cells
BLPC: basal luminal precursor cell
BMP: bone morphogenetic protein
BPD: bronchopulmonary dysplasia
BSC: basal stem cell
COPD: chronic obstructive pulmonary disease
ECFCs: endothelial colony-forming cells
ECM: extracellular matrixDASCdistal alveolar stem cell
ESC: embryonic stem cell
FGF: fibroblast growth factor
Grhl2: Grainyhead-like 2
Hh: hedgehog
hiPSC: human induced pluripotent stem cell
iPSC: induced pluripotent stem cell
Krt: cytokeratin
LNEP: lineage negative epithelial precursor
MSC: mesenchymal stem/stromal cell
NEB: neuroendocrine body
Scgb1a1: secretoglobin family 1a member 1
Sftpc: surfactant protein C
SO_2_: sulfur dioxide
TBSC: tracheal basal cell
TGF-β: transforming growth factor-β
